# Revealing key regulatory factors in lung adenocarcinoma: the role of epigenetic regulation of autophagy-related genes from transcriptomics, scRNA-seq, and machine learning

**DOI:** 10.3389/fphar.2025.1542338

**Published:** 2025-08-04

**Authors:** Xianchang Zeng, Lingyun Wei, Lu Lv, Di Wu, Yingying Shen, Xinliang Lu, Xianghui Kong, Zhijian Cai, Jianli Wang

**Affiliations:** ^1^ Institute of Immunology and Bone Marrow Transplantation Center of the First Affiliated Hospital, Zhejiang University School of Medicine, Hangzhou, China; ^2^ Department of Intensive Care Medicine, Affiliated Hospital of Youjiang Medical University for Nationalities, Key Laboratory of Molecular Pathology in Tumors of Guangxi Higher Education Institutions, Baise, China; ^3^ Department of Orthopaedics of The Second Affiliated Hospital and Institute of Immunology, Zhejiang University School of Medicine, Hangzhou, China; ^4^ Department of Medical Oncology, Zhejiang Cancer Hospital, Hangzhou, China; ^5^ Laboratory of Cancer Biology, Key Lab of Biotherapy in Zhejiang, Cancer Center of Zhejiang University, Sir Run Run Shaw Hospital, Medical School of Zhejiang University, Hangzhou, China

**Keywords:** A-ERGs, LUAD, exhausted CD8^+^T cells., DEGs, machine learning

## Abstract

**Background:**

The molecular pathogenesis of lung adenocarcinoma (LUAD) involves genomic mutations, autophagy dysregulation, and signaling pathway disruptions. Autophagy, a key cellular process, is tightly linked to cancer development; genes like ATG5 and ATG10 influence lung cancer progression, and epigenetic regulators modulate autophagy-related carcinogenesis. However, the role of epigenetic-autophagy genes in LUAD’s tumor microenvironment is under-researched.

**Methods:**

We used the “limma”” package to identify differential epigenetic-related genes associated with altered autophagy regulation (A-ERGs) in LUAD. Single-cell RNA sequencing was further employed to evaluate the heterogeneity of immune cells. Machine learning algorithms were utilized to construct and identify diagnostic markers for LUAD, which were then validated by receiver operating characteristic (ROC) curve analysis. Cell experiments, real-time PCR, and Western blot were conducted to verify the expression of KDM6B and KANSL1 and their effects on T-cell differentiation.

**Results:**

Based on single-cell and transcriptome analyses, we screened 19 A-ERGs that were significantly differentially expressed in lung cancer tissues. These genes were primarily enriched in exhausted T cells. Subsequently, through machine learning, KDM6B and KANSL1 were identified to have excellent diagnostic performance. Single-cell level and transcriptome correlation analyses revealed that the expression of these two genes was associated with exhausted T cells. Results from *in vitro* cell experiments showed that high expression of these two genes promoted the occurrence of T cell exhaustion.

**Conclusion:**

In this study, we utilized bulk and single-cell transcriptomic data to uncover the potential molecular mechanisms of A-ERGs in lung cancer. We explored the characteristic distribution of these genes in the tumor immune microenvironment and identified two A-ERGs, KDM6B and KANSL1, as potential diagnostic biomarkers for lung adenocarcinoma (LUAD). Our findings offer novel strategies for targeted therapeutic interventions in LUAD.

## Introduction

Lung adenocarcinoma (LUAD) is still the most common human malignancy, with high incidence and mortality, which also is the most frequently sub-type of Lung cancer (Sung et al., 2021).LUAD patients are usually diagnosed at an advanced stage and the 5-year survival rate is less than 4%, accounting for 38% of total cases (Dela Cruz et al., 2011). At present, like surgical resection, chemotherapy, and even targeted therapies were mainly treatment for LUAD patients, the prognosis remains poor with low survival rates for the severity of pulmonary fibrosis, high incidence of multi-drug resistances, and diversity of histologic properties (Bade and Dela Cruz, 2020; Tang et al., 2019). The development of LUAD has been shown to be closely related to several factors, such as patients with Chronic obstructive pulmonary disease (COPD)and pulmonary tuberculosis, smoke, immunologic dysfunction, tuberculosis infection; and asthma (Tang et al., 2019; Sekine et al., 2012; Chen et al., 2004). However, there is still poorly information regarding the pathogenic mechanisms driving LUAD initiation and progression. Therefore, it is important to screen novel potential markers of LUAD for predicting the prognosis of individuals with LUAD and serving as therapeutic targets.

The molecular pathogenesis of LUAD is conceptualized as a multi-step process characterized by the progressive accumulation of cellular and molecular alterations, including encompass genomic mutations, Macroautophagy/autophagy, and perturbations in cellular signaling pathways and metabolic processes (Qian et al., 2023; Chen et al., 2024; Guilbaud et al., 2023; Li et al., 2023; Cheng et al., 2025). Macroautophagy/autophagy is a degradative process in which serve as crucial regulators of cellular processes and signaling pathways that drive cancer initiation and progression, playing an indispensable role in the carcinogenic process (Lewerissa et al., 2024). It has been found that the development of lung cancer has been associated with a range of autophagy-related genes (Sharma et al., 2021). For example, low ATG5 expression reduces cell growth in RAS mutant lung cancer cell lines (Guo et al., 2011).ATG10 overexpression was associated with poor prognosis in lung cancer (Honscheid et al., 2014). While a large proportion of cancer carcinogenesis caused by autophagy-related genes was associated with mutations in genes encoding epigenetic regulatory proteins that autophagy-regulate gene expression. Recently, *helicobacter* pylori-induced silencing of MAP1LC3Av1 methylation has been reported to lead to impaired autophagy and promote gastric carcinogenesis (Muhammad et al., 2017). EHMT2 inhibition leads to cancer cell death via autophagy induction in lung cancer (Kim et al., 2020).

There, the above studies suggest that epigenetic-related genes associated with altered autophagy regulation play important roles in tumors and the prognosis of patients with various types of cancer. Although people are developing a growing awareness of the important of the epigenetic regulate autophagy for cancer, little research has focused on the role of these genes in the lung cancer tumour microenvironment. In recent years, the combined analysis of single-cell RNA sequencing (scRNA-seq) and RNA-seq has demonstrated higher sensitivity and accuracy in the study of disease mechanisms. Meanwhile, it has also shown greater efficiency in exploring disease mechanisms (Xie et al., 2024; Zhao et al., 2022; Deng et al., 2024). Therefore, we performed an investigation of epigenetic-related genes associated with altered autophagy regulation (A-ERGs) in LUAD based on transcriptome and single-cell sequencing data. We evaluated the expression of A-ERGs in individuals afflicted with LUAD and their potential correlations to diagnostic, prognostic, and immune infiltration outcomes. Subsequently, through an in - depth exploration of the characteristic distribution patterns of these genes within the tumor immune microenvironment, it was revealed that A- ERG is likely to modulate the oncogenesis of LUAD by mediating exhausted T cells. Two A-ERGs, KDM6B and KANSL1, were identified as potential diagnostic biomarkers for LUAD through machine learning. Our findings contribute to a better understanding of the functional role of A-ERGs in LUAD development and offer insights for the identification of new prognostic markers and therapeutic targets in LUAD.

## Materials and methods

### Acquisition and processing of RNA sequencing (RNA-seq) data

**FIGURE 1:**
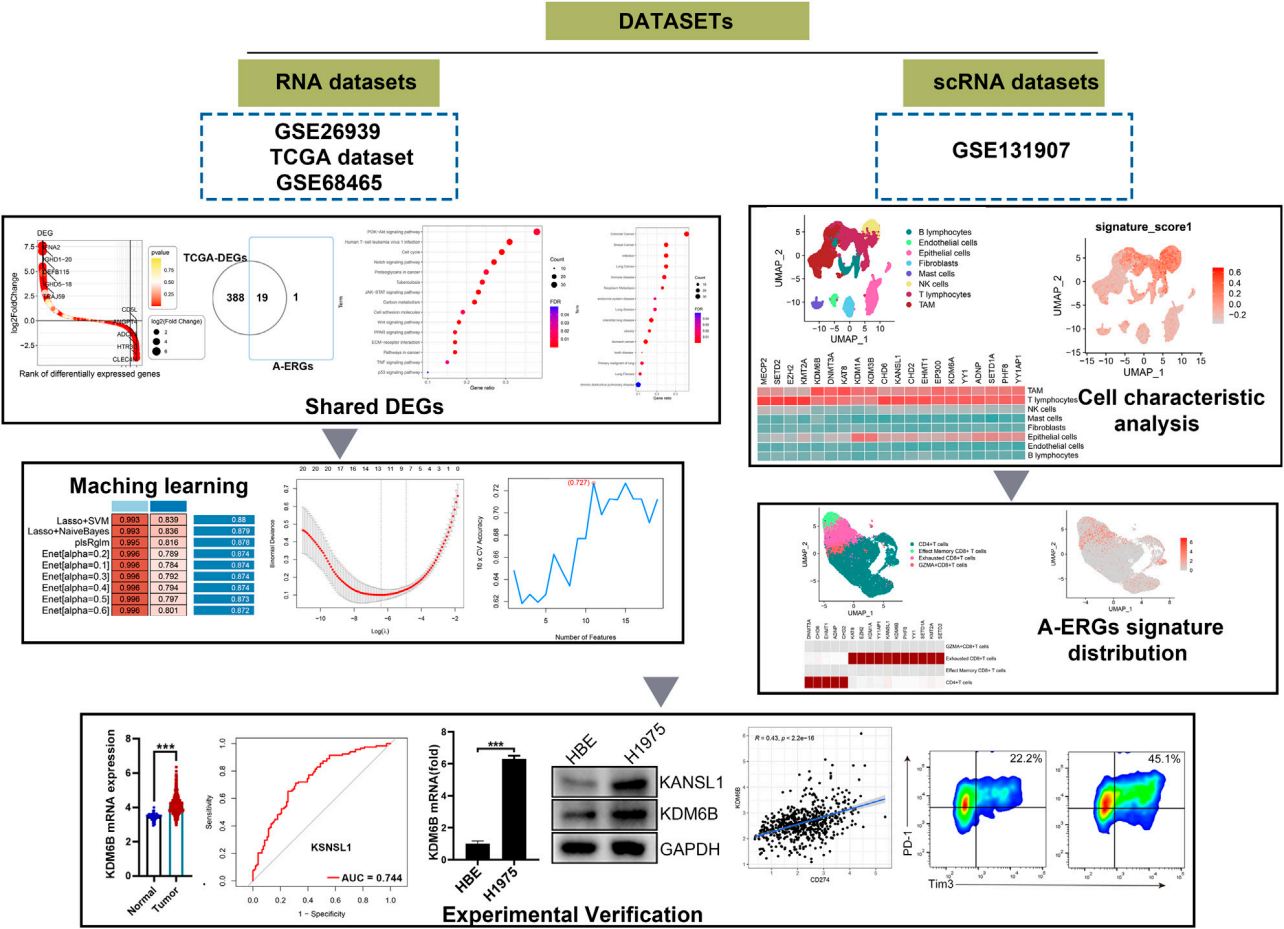
The schematic diagram of this work.

Raw RNA-seq data from 585 LUAD samples (including 58 para-cancerous tissue samples and 527 LUAD samples) and the corresponding clinical data were obtained from the Cancer Genome Atlas (TCGA) database (https://portal.gdc.cancer.gov/). Additionally, other two raw RNA-seq data and clinicopathological information for patients with LUAD (GSE26939 and GSE68465) were downloaded from the GEO database. The GSE26939 expression profile consisting of 116 LUAD tumour samples were processed using the GPL9053Agilent-UNC-custom-4X44K, while the GSE68465 dataset containing 444 LUAD tumour samples were analyzed using the GPL9269 Illumina Genome Analyzer II. The scRNA-seq data of LUAD were download from the GSE131907 data, including 11 distal normal lung tissues and 11 primary LUAD tissues. The gene expression matrix files for the data from all three databases were derived from raw RNA-seq data using R software. Figure 1 provides a flowchart of the overall workflow and study design.

### Differential expression gene analysis

In this study, we utilized the “limma” package (Yin et al., 2022) to identify differentially expressed genes (DEGs) within the LUAD cohort, setting thresholds of |log2FC| > 0.585 and p-value <0.05 (Mayakonda et al., 2018). To visualize the relationship between DEGs and A-ERGs, a Venn diagram was generated using Jvenn (Newman et al., 2015). For the GSE26939 and GSE68465 datasets, the R merge function was utilized to extract and combine these datasets. First, the “limma” package might implicitly assume a certain level of variance among samples during processing. Therefore, data standardization is performed when conducting differential gene analysis on different samples. Following this, we carried out data normalization and DEG screening using the “limma” package, maintaining the same thresholds of |log2FC| > 0.585 and p-value <0.05.

### Functional, disease enrichment analysis and regulatory network construction

To explore the hidden biological characteristics of the shared DEGs and their complex relationships with diseases, analyses including Gene Ontology (GO) annotation and Disease Ontology (DO) assessment were executed via the “cluster profiler” package. Moreover, enrichment analyses for pathways such as the Kyoto Encyclopedia of Genes and Genomes (KEGG) Pathways, and WikiPathways were conducted using Enrichr (https://maayanlab.cloud/Enrichr/).

### Analysis of single-cell RNA sequence data

The expression matrix was normalized using the Seurat software package (version 4.3.2, accessible at https://satijalab.org/seurat/), which also allowed for the generation of scaled data by factoring in the UMI counts from each sample and the proportion of mitochondrial gene expression. Cells of insufficient quality were filtered out according to specific thresholds: <200 genes per cell, <10 cells associated with each gene, and a mitochondrial gene expression rate >15%. After this filtering process, the top 2,000 highly variable genes (HVGs) were selected for integration of the samples using the “FindVariableGenes” function, followed by principal component analysis of these HVGs carried out through the “RunPCA” function (Zou et al., 2023). To correct for batch effects, present across the various samples, the “Harmony package” was employed (Cao et al., 2023). Subsequently, we established a resolution of 1.0 to facilitate the identification of cell types across all populations, projecting the cells into a two-dimensional representation using the “RunUMAP” function. To illustrate the cell clusters, the “Dimplot” function was used (Huang et al., 2021). The “UCell” package was then utilized to compute enrichment scores for A-ERGs throughout the cell population (Andreatta and Carmona, 2021). For the identification of DEGs across various groups in same cell types, a differential expression analysis was conducted using the FindMarkers function available in Seurat. The criteria for DEG identification were defined as follows: |average fold change| ≥ 0.25 and P-value ≤0.05.

### Construction of diagnostic model

We utilized a total of ten varied machine learning algorithms for integration, encompassing methods such as Least Absolute Shrinkage and Selection Operator (LASSO), Gradient Boosting Machine (GBM), Random Survival Forest (RSF), Partial Least Squares Regression for Cox (plsRcox), Stepwise Cox (StepCox), Supervised Principal Components (SuperPC), Ridge Regression, Survival Support Vector Machine (Survival-SVM), CoxBoost, and Elastic Network (Enet). An evaluation of 101 different combinations of these algorithms was conducted (Liu et al., 2022; Reel et al., 2021). Our approach followed a sequenced strategy, which included identifying prognostic factors through univariate Cox regression, creating predictive models based on the TCGA-LUAD dataset, and validating these models against additional independent datasets (GSE26939 and GSE68465). The Harrell Consistency Index (C-index) was then calculated to facilitate model selection. The optimal model was determined to be the one that achieved the highest average C-index across all datasets.

### ROC curve

To thoroughly examine the diagnostic model for LUAD, we performed receiver operating characteristic (ROC) curve analyses on the gene expression datasets TCGA-LUAD cohort, using the pROC package (Robin et al., 2011). At the same time, we executed ROC curve analyses on the GSE26939 and GSE68465 datasets to evaluate the diagnostic potential of key genes related to LUAD. The area under the curve (AUC) was utilized to compare the ability of the LASSO models to diagnose LUAD with the diagnostic effectiveness of individual LUAD-associated genes.

### Cell culture

Human LUAD cell lines H1975, and mouse Lewis lung carcinoma (LLC) were purchased from the American Type Culture Collection. All cell lines were cultured in DMEM (Thermo Fisher Scientific), supplemented with 10% (v/v) foetal bovine serum (FBS) and 100 μg/mL penicillin–streptomycin. All cells were maintained at 37°C and 5% CO2 in a humidified incubator.

### RNA isolation and quantative PCR (qPCR)

Total RNA was extracted using the TRIzol reagent (China, shanghai; Thermo Fisher Scientific) as per the manufacturer’s protocol. A reverse Transcription kit (Takara) was used to synthesise cDNA and a SYBR Green Master Mix kit (Vazyme, Q221-01) was used to perform qPCR on the Roche LightCycler^®^ 480II platform (Roche Diagnostics, United States). The PCR procedure was as following: 1 cycle at 95°C for 30 s, followed by 40 amplification cycles of denaturation at 95°C for 5 s and annealing extension at 60°C for 34 s. The primers used for the qPCR were listed in Table 1. The fold changes of gene expression levels were calculated using the Formula 2^−ΔΔCt^.

**TABLE 1:** qPCR Primer of KDM6B and KANSL1.

KDM6B-F	AGA​CCT​CAC​CAT​CAG​CCA​CTG​T
KDM6B-R	TCT​TGG​GTT​TCA​CAG​ACT​GGG​C
KANSL1-F	TGC​CAT​GCA​GTC​TGT​CAG​ATC​C
KANSL1-R	CAA​GTA​GCT​CGG​ACT​GCT​CAT​G

### Western blotting

A total of 10 μg cell lysates were resuspended with 5 × SDS loading buffer and subsequently incubated at 100°C for 5 min. After centrifugation, the supernatants were separated by a SDS-PAGE and transferred onto a PVDF membrane (Millipore). The proteins-imprinted membranes were blocked with 5% bovine serum albumin for 1 h and incubated with the corresponding primary antibodies at 4°C overnight. After washed thrice, the membranes were incubated with horseradish peroxidase-coupled secondary antibodies for 1 h at room temperature, each membrane was scanned using a Tanon 4500 imaging system (Shanghai, China).

### RNA interference

mRNA mimics and negative control (NC) were designed and purchased from GenePharmach Company (shanghai, China). Transient transfections of these mimics into LLC, A549, and H1975 cells were performed with the INTERFERin^®^ Transfection Reagent (Polyplus Transfection) at a final concentration of 20 nM, according to the manufacturer’s instructions. Cells were harvested 48 h after transfection for qPCR and Western blotting analyses.

### Statistical analysis

Statistical analyses were performed with GraphPad Prism 8.0 software. The differences between two groups were analyzed by unpaired Student’s t-test, and differences among multiple groups were analyzed by one-way or two-way ANOVA followed by the Tukey test. The log-rank test was used for survival analysis, and the Spearman rank-order correlation test was used for Pearson correlation analysis. A difference was considered significant if the p value was <0.05.

## Results

### Identification of differentially expressedA-ERGs in LUAD samples

**FIGURE 2:**
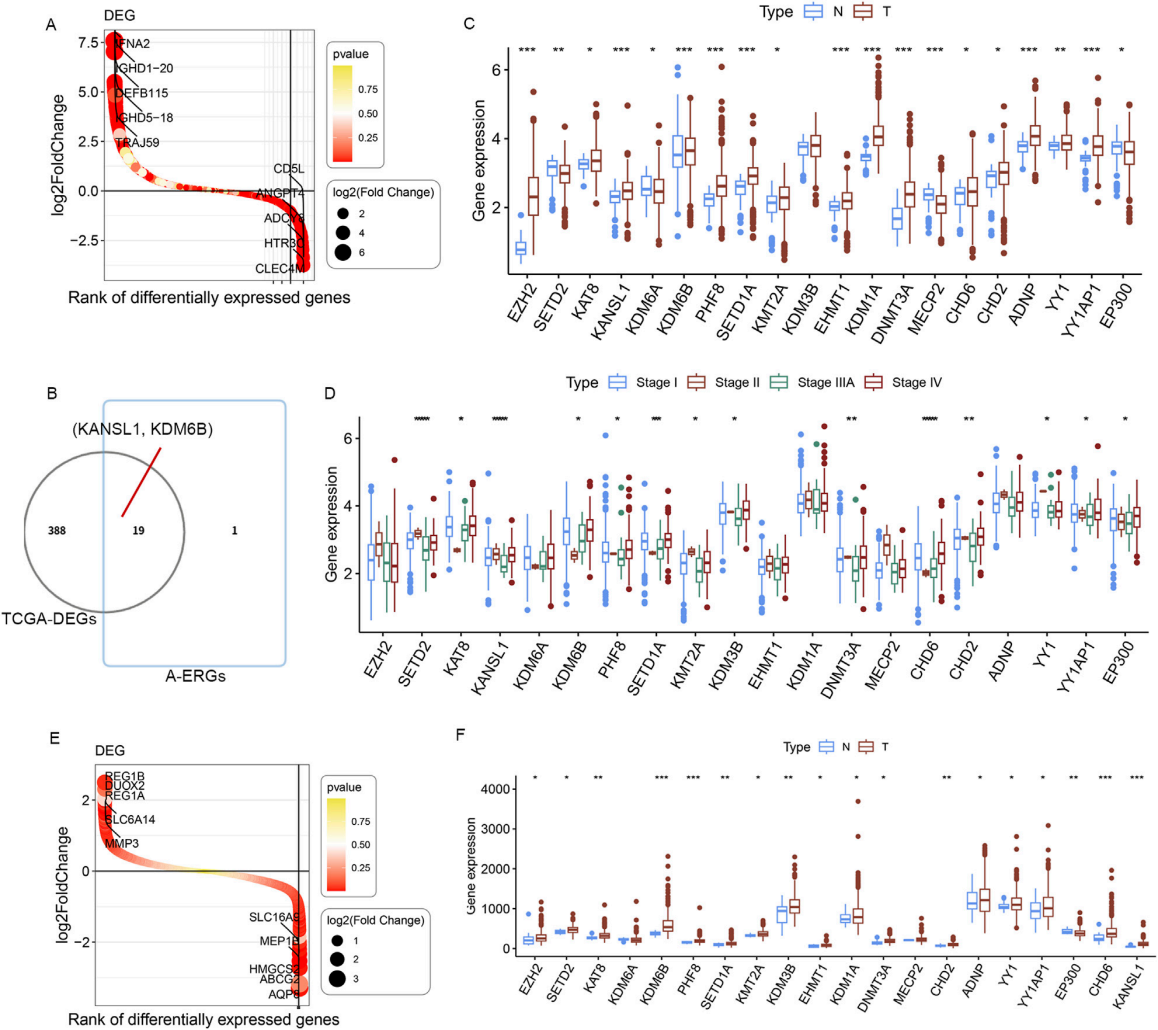
Identification of differentially expressedA-ERGs between normal and LUAD samples. **(A)** Waterfall plot of DEGs between normal and LUAD in TCGA dataset. Red and yellow represent the magnitude of the *p*-value. **(B)** The Venn diagrams illustrate the common genes between DEGs and A-ERGs. **(C)** The expression of these 19 A-ERGs between normal tissues and LUAD tissues in TCGA dataset. Red, tumor sample; blue, normal sample.**(D)** The expression analysis of 19 A-ERGs in different stages of LUAD. **(E)** Waterfall plot illustrate the DEGs between normal and LUAD in GSE26939 and GSE68465 datasets. **(F)** The expression of these 19 A-ERGs between normal tissues and LUAD tissues in GSE26939 and GSE68465 datasets.

The literature search (Lewerissa et al., 2024) identified a total of 20 epigenetic genes that regulate autophagy (A-ERGs), including Histone modifiers (EZH2, SETD2, KAT8, KANSL1, KDM6A, KDM6B, PHF8, SETD1A, KMT2A, KDM3B, EHMT1 and KDM1A), DNA methyltransferases (DNMT3 and MECP2), Chromatin remodelers (ADNP, YY1 and YY1AP1) and Cytoplasmic protein modifiers (EP300, EHMT1 and SETD2). Using the R package limma, we analyzed the DEGs in TCGA-LUAD cohort, and found that most of these genes showed significant differences in lung cancer tissues (Figures 2A,B). Next, we visualized the differential expression of these A-ERGs in normal and lung cancer samples using boxplots. The results showed that there are genes with different expression trends among these A-ERGs. For example, the EZH2, KDM1A, KANSL1, KDM6B, and KAT8 genes are upregulated in lung cancer patients, while EP300, MECP2, and SETD2 are downregulated in lung cancer (Figure 2C). Considering the important biological functions of these genes in tumorigenesis and development, we systematically studied the relationships between these regulatory factors and the pathological characteristics of lung cancer. The results showed that most genes, including SETD2, KAT8, KANSL1, KDM6B, PHF8, SETD1A, KDMT2A, KDM3B, DNMT3A, CHD6, CHD2, YY1, YY1AP1 and KDM1A were significantly correlated with the grading of lung cancer patients (Figure 2D). Subsequently, we further analyzed the expression levels of these genes in lung cancer tissues using other external validation datasets (GSE68465 and GSE26939). Consistent with the previous results, most genes showed significant differences in lung cancer (Figures 2E,F). Therefore, these data indicate that these A-EGRs may play an important role in the process of lung cancer development.

### GO, DO, and pathway enrichment of the differentially expressedA-ERGs

To reveal the potential biological processes, molecular functions, and related diseases of these differentially expressed A-ERGs, we explored the enriched pathways of these differentially expressedA-ERGs in two databases (KEGG and Wikipathway). Pathway enrichment analysis showed that these genes were significantly involved in the PI3K-AKT signaling pathway, JAK-STAT signaling pathway, TNF signaling pathway, p53 signaling pathway, TGF-beta signaling pathway, PPAR signaling pathway, ECM-receptor interaction and Notch signaling pathway (Supplementary Figures S1A, S1B). These data indicate that these genes are mainly involved in immune-related signaling pathways. Meanwhile, the DO analysis revealed that these A-ERGs were markedly enriched in lung disease, immune disease, lung cancer, obesity, endocrine system disease and stomach cancer (Supplementary Figure S1C).

### High expression of the A-ERGs in T cells of LUAD patients

**FIGURE 3:**
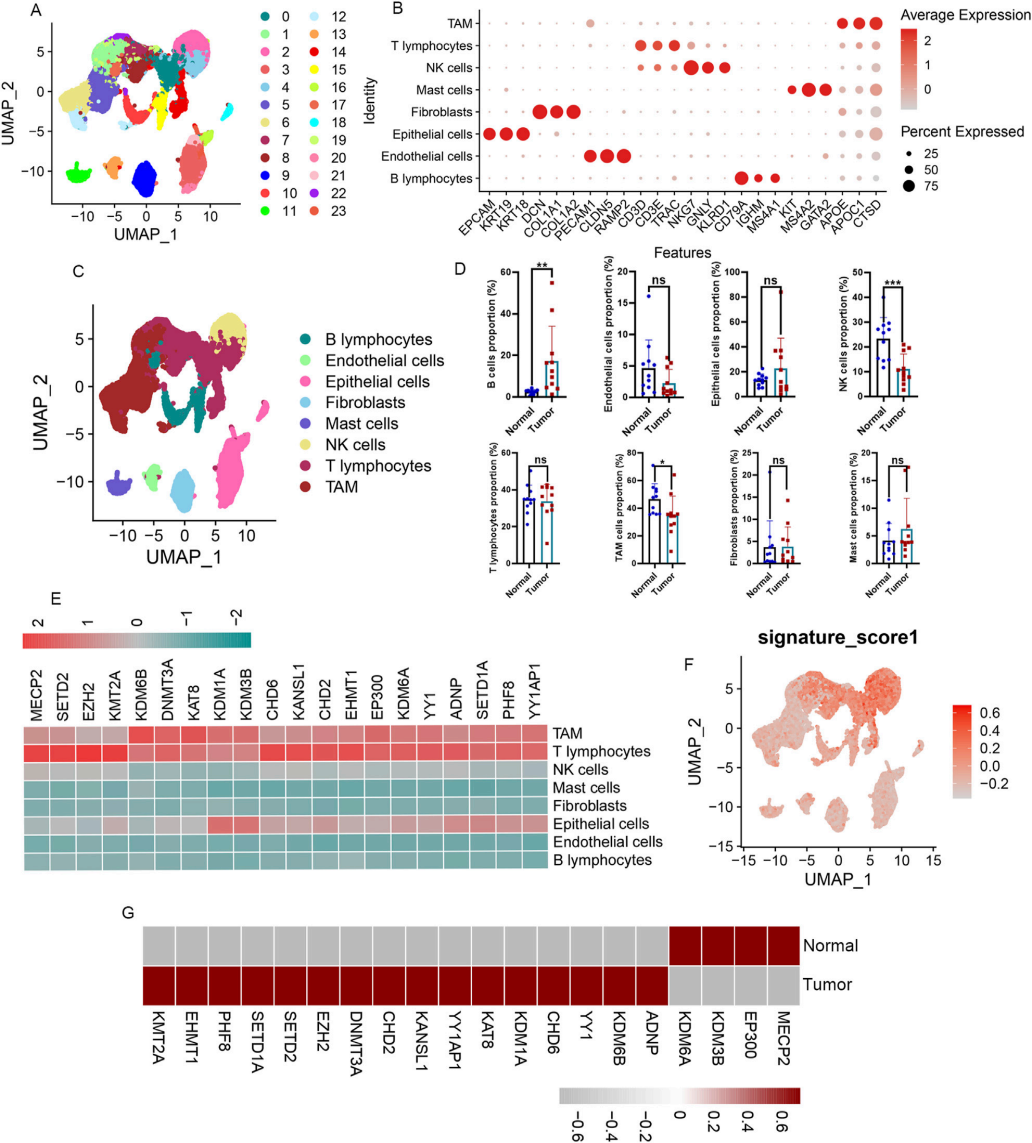
Immunological characterization of differentially expressed A-ERGs in the single cell atlas of LUAD patients. **(A)** UMAP display of the clusters of immune cells in LAUD patients. **(B)** The dot plot presents the annotation markers pertaining to the cell clusters. **(C)** UMAP plots showing the main cell types of LUAD samples. **(D)** Comparison of cell type proportions between normal and tumor groups. **(E)** Heatmap visualization of the expression patterns of these differentially expressed A-ERGs in each cell type. **(F)** Module score distribution in UMAP space for these differentially expressed A-ERGs modules was evaluated using “UCell” in different cell types. **(G)** Heatmap visualization of the expression patterns of these differentially expressed A-ERGs in different cell subgroups. *p < 0.05; **p < 0.01; ***p < 0.01; ns, not significant.

To deeply explore these A-ERGs’expression profiles across different cell types in LUAD, we performed scRNA-seq analysis. A total of 63,314 cells were analyzed from the 22 samples (including 11 patients with LUAD, and 11 healthy controls), 24 clusters were found among the cells using a graph-based clustering technique coupled with the uniform manifold approximation and projection (UMAP) dimensionality reduction method (Figure 3A). We then identified 8 cell types based on the classical markers (Figures 3B,C; Supplementary Figures S2A, S2B), including B cells, T cells, Endothelial cells, Epithelial cells, Fibroblasts, Mast cells, Tumour-associated macrophages (TAM), and natural killer (NK) cells. We observed a significant increase in the proportion of B cells among LUAD patients when compared to healthy controls (Figure 3D; Supplementary Figure S2C). Conversely, the proportions of TAM cells and NK cells were reduced in the LUAD cohort (Figure 3D; Supplementary Figure S2C). To deeply investigate these A-ERGs expression in these cells, we utilized the “UCell” package to assess module scores based on 19 A-ERGs. In LUAD patients, T cells demonstrated the highest module scores, aligning with heatmap data that emphasized their elevated expression of these A-ERGs (Figures 3E,F). In addition, we also assessed the expression levels of these A-ERGs in T cells across all samples and found that, compared to the control group, the expression of most A-ERGs was consistently higher in the lung cancer group, except for KDM3B, EP300, MECP2, and KDM6A (Figure 3G). These findings suggest that the activity of these A-ERGs in T cells may be associated with the development of LUAD and the dysfunction of T cells in the tumor micro-environment.

### Increased proportion and A-ERGs expression in exhausted CD8^+^T in LUAD patients

**FIGURE 4:**
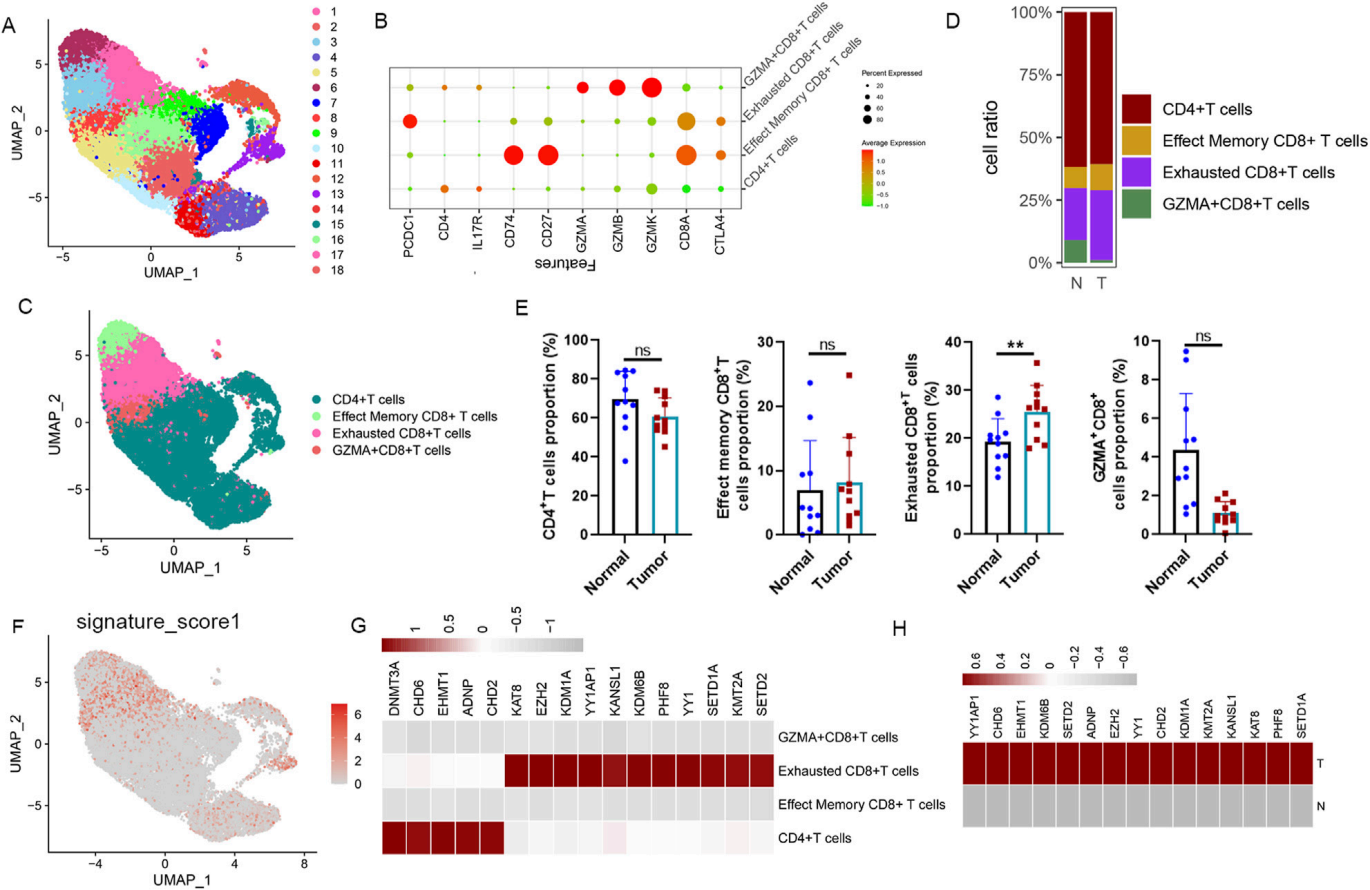
ScRNA-seq reveals the immunological properties of differentially expressed A-ERGs in T cell subgroups. **(A)** UMAP plot showing each cluster of T cell subgroups, coloured by different clusters. **(B)** A dot plot visualization the expression of marker genes in different clusters. **(C)** The UMAP plot shows each cell type of T cell subgroups, colored by different cell types. **(D)** The Bar chart shows the relative frequency of each subgroup of T cells in different groups. **(E)** Comparison of each subgroup of T cells in different groups. **(F)** The distribution of module scores of differentially expressed A-ERGs modules in the UMAP space was evaluated in different cell types. **(G)** The heatmap illustrates the expression distribution of these differentially expressed A-ERGs within each subpopulation of T cells. **(H)** Heatmap visualization of the expression patterns of these differentially expressed A-ERGs in different cell subgroups. **p < 0.01; ***p < 0.01; ns, not significant.

To further elucidate the influence of these A-ERGs on T cells in patients with LUAD, we conducted a subpopulation analysis of T cells, examining the distribution of upregulated A-ERGs across T cell subgroups. Using UMAP analysis, T cell profiles were categorized into 19 distinct cellular clusters (Figure 4A). This clustering revealed four primary cell types, including CD4^+^T cells, Effected Memory CD8^+^T monocytes, GZMA^+^CD8^+^T cells and Exhausted CD8^+^ T cells, identified through classical markers (Figures 4B,C). Concurrently, we measured the variations in proportions of these cell types, finding a decrease in the number of CD8^+^ T cells among LUAD patients and a notable rise in the proportion of exhausted CD8^+^T cells (Figures 4D,E). Conversely, the proportions of GZMA^+^CD8^+^T cells were reduced in the LUAD cohort (Figures 4D,E). Following this, we utilized the “UCell” software package to assess the module scores of the 16 upregulated A-ERGs across different cell types. The analysis revealed that exhausted CD8^+^T cells in the LUAD cohort exhibited the highest module scores, as visualized through UMAP, significantly surpassing those in the control group (Figure 4F). Moreover, heatmap analysis further confirmed that these A-ERGs were predominantly enriched in exhausted CD8^+^T cells, displaying elevated expression levels in LUAD patients (Figures 4G,H). Collectively, these findings indicate that A-ERGs are highly expressed in depleted T cells of LUAD patients and may play a role in exacerbating the state of these patients by driving T cell dysfunction.

### Identification of biomarkers for LUAD by machine learning

**FIGURE 5:**
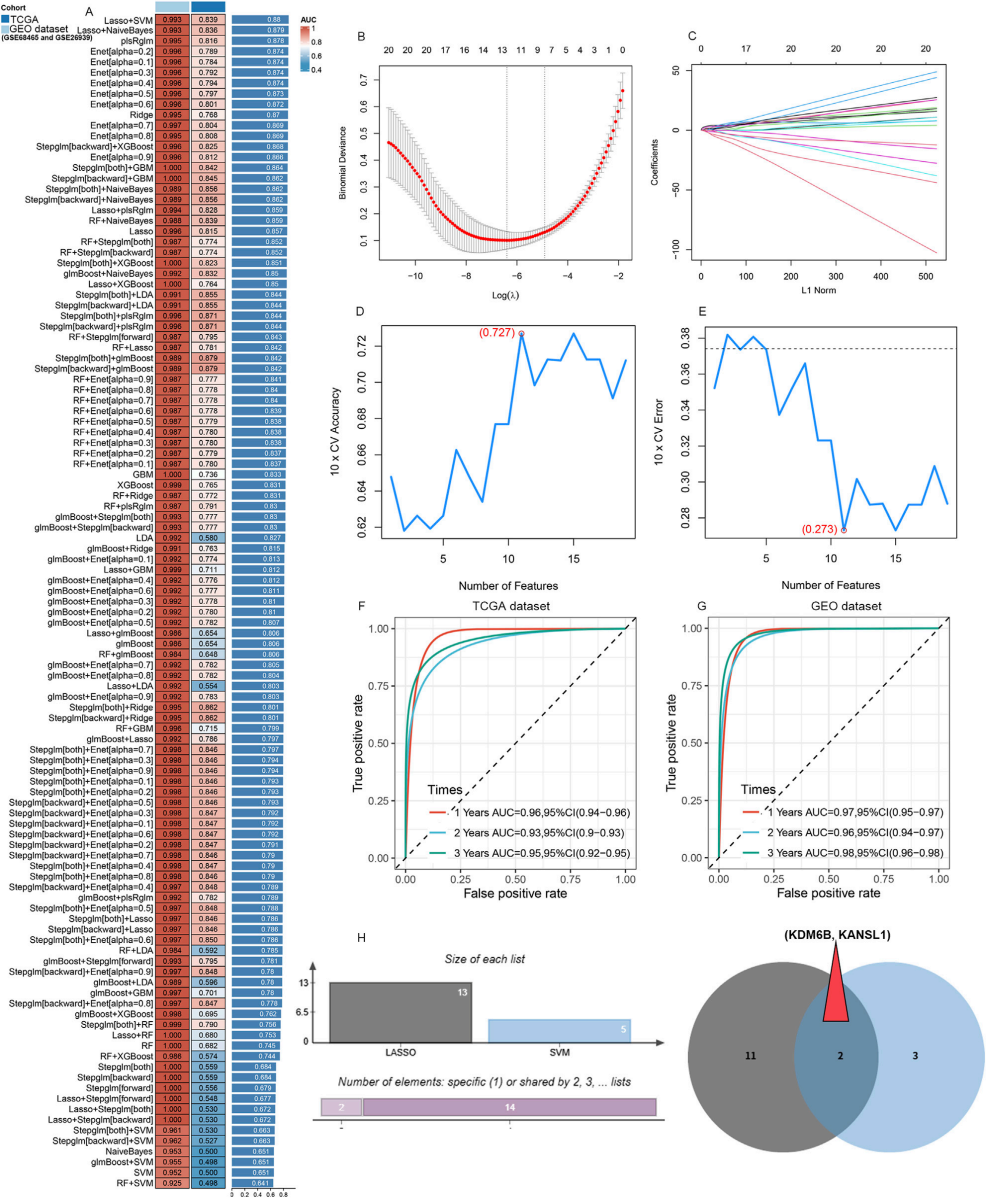
Candidate diagnostic biomarkers for LUAD are being screened by machine learning. **(A)** The C-index of multiple models derived from combinations of different machine learning algorithms in three cohorts (TCGA dataset, GSE68465 and GSE26939). **(B)** When performing LASSO regression on the predictive genes, the plot clearly illustrates the Lasso coefficients for each gene in association with the logarithmically scaled λ (lambda) value. **(C)** Mean Square Error (MSE) penalty plot obtained through cross - validation for the Lasso model. **(D,E)** Feature genes screening using the Support Vector Machine - Recursive Feature Elimination (SVM - RFE) algorithm. **(F,G)** ROC curve analysis of the C index of the Lasso joint SVM over time at 1-year, 2‐years and 3‐years survival in the training cohorts. **(H)** A Venn diagram shows that two candidate diagnostic genes are recognized through the two algorithms.

To develop a robust predictive model, we utilized selected genes as input features and assessed ten machine learning methodologies: Random Survival Forest (RSF), Elastic Net (Enet), stepwise Cox regression, CoxBoost, Partial Least Squares Regression for Cox (plsRcox), Lasso regression, Ridge regression, SuperPC, Gradient Boosting Machine (GBM), and survival-support vector machine (survival-SVM). Utilizing the TCGA-LUAD dataset as our test dataset, along with other external validation datasets (GSE26939 and GSE68465), we compared the concordance index (C-index) of these models. Among them, RSF emerged as the leading model, demonstrating superior C-index performance (Figure 5A). An optimal diagnostic signature was constructed using a combination of Lasso and SVM-RFE algorithms (Figures 5B–E). We subsequently validated the model through Receiver Operating Characteristic (ROC) analysis. For the training TCGA cohort, the Area under the curve (AUC) was 0.993 (Supplementary Figures S3A, S3C); the test datasets GSE68465 exhibited AUCs of 0.838 (Supplementary Figures S3B, S3D), respectively. Meanwhile, for the training cohort of LUAD patients, the area under the curve (AUC) exceeded 0.96, 0.93 and 0.95 for the 1-year, 2-year, and 3-year separately (Figure 5F). The test GSE6846 dataset, exhibited AUC values of 0.97, 0.96, and 0.98 at the 1-year, 2-year, and 3-year time points (Figure 5G). Subsequently, KDM6B, and KANSL1 as the top two important features among these A-ERGs via these two algorithms (Figure 5H). These observations indicate that KDM6B and KANSL1 possess significant diagnostic value in the pathological process of aggravated lung cancer.

### Validation of KANSL1, KDM6B expression in different lung cancer cell lines

**FIGURE 6:**
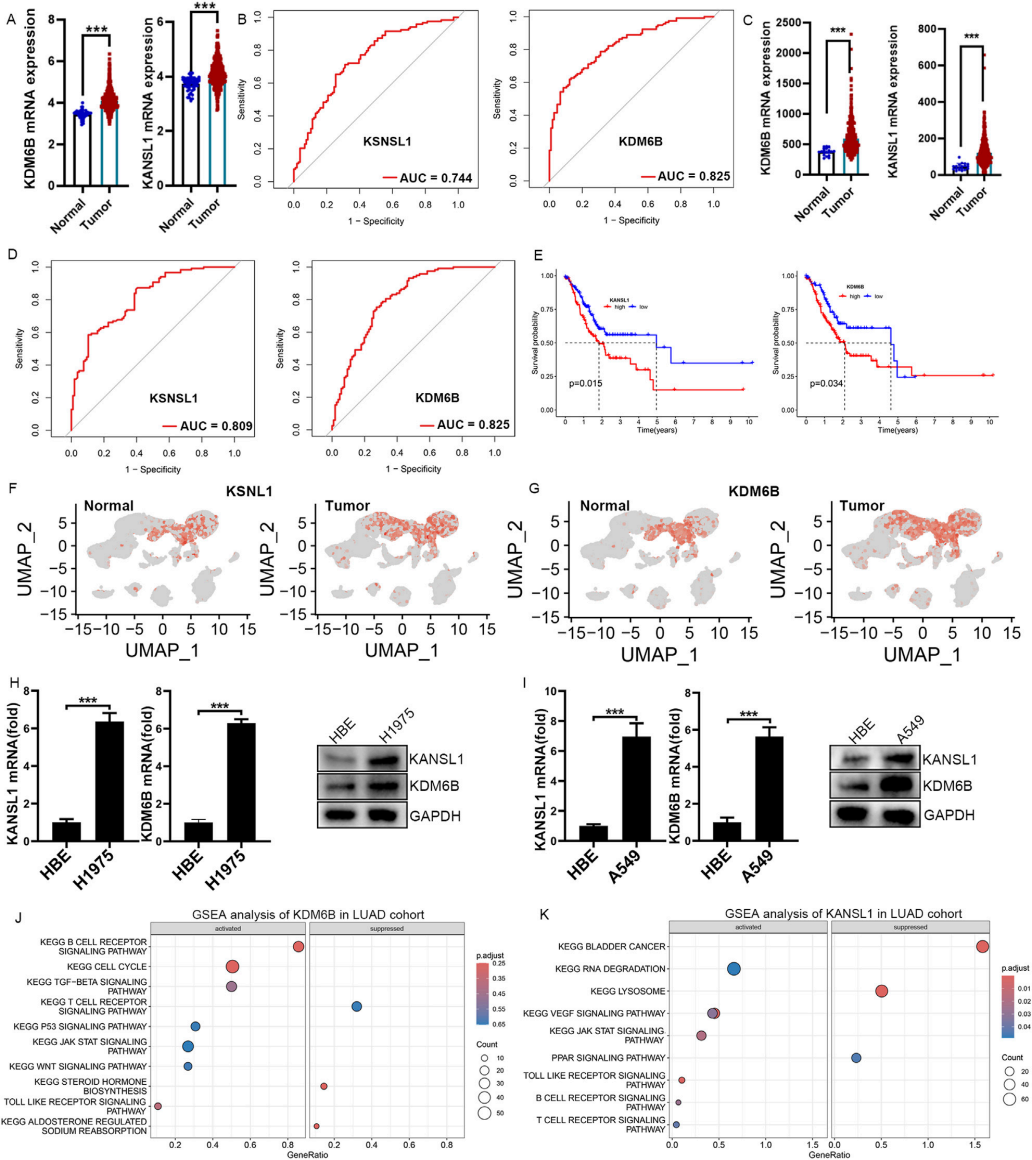
Real-Time qPCR Confirms Elevated KDM6B and KSNSL1 mRNA Levels in LUAD cells. **(A,C)** The difference in mRNA expression levels of KSNSL1 and KDM6B in LUAD patients, based on datasets TCGA and GSE68465. **(B,D)** ROC curve analysis of KSNSL1 and KDM6B in LUAD patients in TCGA and GSE68465. **(E)** Survival analysis of these two A-ERGs in the TCGA dataset. **(F,G)** UMAP plot showing the expression levels of KSNSL1 and KDM6B in different cell types. **(H,I)** The results of the qPCR and Western blot confirmed the mRNA and protein levels of BCL2A1 and CEBPB in LUAD cells. **(J,K)** GSEA function enrichment analysis of KSNSL1 and KDM6B in LUAD patients. ***p < 0.001.

To explore the mRNA expression levels of KDM6B and KANSL1, we consulted datasets from healthy individuals as well as patients with LUAD. The expression level of these two genes were significantly upregulated in patients compared with controls (Figure 6A). Additionally, the ROC analysis confirmed that the expression of KDM6B, and KANSL1 were of great diagnostic value in LUAD patients (Figure 6B). Meanwhile, additional datasets (GSE26939 and GSE68465 and GSE118370) further confirmed the high expression of these two genes in LUAD patients (Figures 6C,D; Supplementary Figures S4A, S4B). Subsequently, the prognostic value of these two genes was further evaluated, and it was found that the high expression of these two genes was significantly associated with the poor prognosis of lung cancer (Figure 6E). Besides,the results of single-cell transcriptome analysis also showed that these two genes are highly expressed in CD8^+^T cells of LUAD patients (Figures 6F,G). In addition, human A549 LUAD cells, and mouse LLC LUAD cells had notably higher mRNA and protein levels of KDM6B, and KANSL1 than normal pulmonary epithelial cells (Figures 6H,I). We therefore infer that these two genes play an essential role in the occurrence and development of LUAD. To further explore the potential biological functions of these two genes in LUAD, we conducted GSEA enrichment analysis at the transcriptomic level. In the LUAD cohort, the GSEA enrichment analysis of KDM6B highlighted seven activated KEGG gene sets and three repressed KEGG gene sets, with the JAK-STAT. TGF-beta signaling pathway, B cell receptor signaling pathway, p53 signaling pathway and Toll like receptor signaling pathways significantly upregulated, while the T cell receptor signaling pathway and Steroid hormone biosynthesis was downregulated (Figure 6J). Remarkably, the GSEA enrichment analysis results for KANSL1 demonstrated that seven pathways—the Vegf signaling pathway, B cell receptor signaling pathway, Toll like receptor signaling pathway and JAK-STAT signaling pathwa, were enriched and significantly upregulated in LUAD cohorts (Figure 6K). These overlapping pathways indicate that KDM6B and KANSL1 might facilitate the development of LUAD via comparable pathological mechanisms.

### Systematic immune characteristics of KANSL1and KDM6B in LUAD patients

**FIGURE 7:**
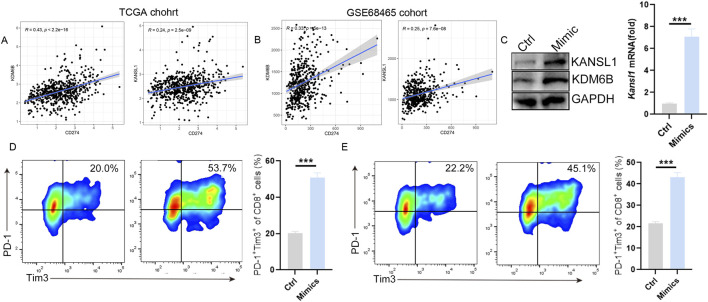
KDM6B and KANSL1 induce the exhaustion of CD8^+^ T cells. **(A,B)** Correlation analysis between PD-L1(CD274) and KDM6B and KANSL1 in LUAD, based on TCGA cohort and GEO cohort. **(C)** Detect the expression levels of KDM6B and KANSL1 genes and proteins after treatment with mimics by qPCR and Western blot. **(D)** After the overexpression of KDM6B and KANSL1, perform flow cytometric analysis *in vitro* to determine the proportion of exhausted CD8^+^T cells.

The above single-cell study showed that exhausted CD8^+^T cells in LUAD patients increased significantly. To further investigate whether these two prognostic markers are associated with T-cell infiltration in the pathogenesis of LUAD, we conducted a Pearson correlation analysis between these two genes (KDM6B and KANSL1) and immune cells. Consistent with the single-cell results, in the LUAD group, the infiltration level of CD8^+^T cells decreased significantly, while that of M2 macrophages increased significantly (Supplementary Figures S5A, S5B). Meanwhile, functional analysis further demonstrated that the functions of CD8^+^T cell infiltration and the cytolytic activity significantly decreased in the LUAD group as well (Supplementary Figure S5C). Besides, the survival analysis showed that the decrease in the number and function of T cells was significantly associated with the poor prognosis of LUAD (Supplementary Figure S5D). Notably, in line with the single-cell results, the expressions of KDM6B and KANSL1 are significantly negatively correlated with CD8^+^T cells and Gamma delta^+^ T cells in LUAD (Supplementary Figure S5E) and are significantly positively correlated with the expression of PD-L1 (Figures 7A,B). To further clarify the impact of these two genes on the function of T cells, we conducted *in vitro* cell experiments. The results showed that the increased expression levels of KDM6B and KANSL1 significantly increased the proportion of exhausted CD8^+^T cells (Figures 7C–E). Thus, the above results suggested that these two genes may promote the progression of LUAD through riving T cell dysfunction.

## Dicussion

LUAD is a highly heterogeneous and invasive disease with a low overall survival rate, and it is prone to tumour recurrence and metastasis after treatment. The occurrence of lung cancer is closely related to a series of genetic alterations (Denisenko et al., 2018; Shi et al., 2016). In recent years, with the continuous development of sequencing technologies and bioinformatics, molecular diagnosis and molecular therapy have gradually attracted increasing attention in the field of oncology. Autophagy is a highly conserved catabolic pathway, which plays a crucial role in maintaining the stability of the intracellular environment. Alterations in autophagy-related genes are associated with the prevalence and progression of lung cancer (Mizushima and Komatsu, 2011; Kroemer et al., 2010). Clinical studies have shown that the overexpression of p62 is related to the overall survival of lung cancer patients (Wang et al., 2019). In addition, in the mouse model bearing lung cancer, inhibiting caspase-3 and upregulating autophagy via mTOR can enhance the efficacy of radiotherapy (Li et al., 2019). The precise transcription of genes involved in autophagy is regulated by a network of epigenetic factors. Epigenetics is generally regarded as the heritable variation in gene expression or phenotype, rather than changes in the DNA sequence. The transcription factor Forkhead box O3 (FOXO3) and the surface marker CD47 can both regulate the occurrence of autophagy (Wang et al., 2016; Li et al., 2018). It has been reported that aberrantly expressed HDAC8 promotes the occurrence of oral squamous cell carcinoma by activating caspase - induced apoptotic cell death and promoting autophagy (Ahn and Yoon, 2017a). Silencing of HDAC7 inhibits salivary mucinous epidermoid carcinoma cytogenesis by inducing apoptosis and autophagy (Ahn and Yoon, 2017b). Lysine specific demethylase 1 (LSD1) plays an important role in the treatment of neuroblastoma and acute myeloid leukemia (AML) by mediating p62 expression (He et al., 2020). Although there is a growing awareness of the importance of epigenetic regulation of autophagy in cancer, the role of these autophagy - related epigenetic genes in lung cancer, especially in the tumor microenvironment, remains unclear.

In this study, we developed a multi-omics approach integrating transcriptomics, scRNA-seq, and machine learning to investigate the expression profiles of DEGs related to the epigenetic regulation of autophagy-related genes (A-ERGs) in LUAD. We also determined the roles of potential molecular targets in the immune microenvironment of lung cancer and their potential associations with the occurrence of the disease. Here, we identified the DEGs in the A-ERGs that are significantly expressed in LUAD and discovered the biological functions of these DEGs in the pathogenesis of LUAD. Interestingly, these A-ERGs are significantly enriched in PI3K-AKT signaling pathway, JAK-STAT signaling pathway, TNF signalling pathway, p53 signalling pathway, TGF-beta signalling pathway, PPAR signalling pathway, ECM-receptor interaction and Notch signaling pathway.

To determine the expression landscape of these A-REGs in different immune cells, we further investigated the characterization of the single-cell profiles of LUAD samples. We found that, compared with the control group, the numbers of B cells, epithelial cells, and tumor-associated macrophages in tumor cells increased significantly, while the number of natural killer (NK) cells decreased significantly. There was no obvious difference in the number of T cells between two groups. Surprisingly, most of the A-ERGs are enriched in T cells, and their expression levels are higher than those in the control group. The changes in the activity of T cells play an important role in tumor progression. Therefore, we can infer that these hub genes may be involved in the occurrence of tumors by regulating the function of T cells. To further analyze the potential mechanisms of these A-ERGs in the function of T cells, we further studied the cellular profile characteristics of T cell subsets in LUAD. We emphasize that in LUAD, the number of exhausted CD8^+^T cells has significantly increased, while the number of effectors CD8^+^T cells has significantly decreased. The spatiotemporal exhaustion of cytotoxic CD8^+^ T cells within the tumor microenvironment (TME) promotes tumor escape (Song et al., 2022). Inhibitory molecules such as cytotoxic T lymphocyte-associated protein 4 (CTLA-4) and programmed cell death protein 1 (PD-1) exist in cytotoxic CD8^+^T cells within the tumor microenvironment (TME), leading to poor clinical prognosis in LUAD patients (Zhang et al., 2021). Interestingly, we found that these A-ERGs were mainly enriched in exhausted CD8^+^T cells of LUAD patients. In patients with LUAD, the response of CD8^+^T exhausted cells may be the main cause of the progression of LUAD. Therefore, we infer that these A-ERGs may promote the transformation of T cells into exhausted T cells, thereby triggering tumor immune escape.

We then used LASSO regression and SVM-RFE machine learning to screen out two A-ERGs as candidate biomarkers for LUAD. KDM6B and KANSL1 were identified as potential biomarkers with diagnostic value for LUAD. Two genes are significantly upregulated in human LUAD disease. Lysine-specific demethylase 6B (KDM6B), is a key histone demethylase in various normal and pathological processes such as inflammation, development, aging, and cancer (Salminen et al., 2014).High levels of KDM6B have been confirmed to regulate tumor progression by mediating cell proliferation, migration, and senescence (Cheng et al., 2025; Xun et al., 2021). TGF-β (transforming growth factor β1) is an inducer of EMT (epithelial-mesenchymal transition) and tumor metastasis. Knocking down KDM6B can inhibit the invasion of breast cancer cells by suppressing TGF-β-induced EMT (Ramad et al., 2020). Another study has also confirmed that in glioblastoma cell lines, KDM6B is involved in cell proliferation, migration, and invasion by inducing the expression of SNAI1 (Salminen et al., 2014).In addition, the deletion of Kdm6b enhances antigen presentation, interferon response, and the efficacy of ICI (immune checkpoint inhibitor) immunotherapy in myeloid cells by suppressing immunosuppressive mediators, including Mafb, Socs3, and Sirpa (Goswami et al., 2023). KAT8 regulatory NSL complex subunit 1(KANSL1), encodes a widely expressed nuclear protein, which is a member of the non-specific lethal (NSL) complex and is in the q21.31 region of chromosome 17 (Dingemans et al., 2021). KANSL1 has been confirmed to be essential for the acetylation of p53 at lysine 120 (K120), thereby regulating the transcriptional activation of p53 target genes, which is an important activator in tumorigenesis and metastasis (Li et al., 2009). The KANSL1 gene encodes a nuclear protein involved in chromatin modification and has been reported and confirmed to be a cancer driver gene participating in epigenetic modification (Chang et al., 2019). Besides, KANSL1 is amplified and rearranged in ovarian cancer, and the overexpression of its mRNA is a highly predictive indicator of poor prognosis (Fejzo et al., 2021). Interestingly, high expression of KANSL1 can lead to a shift in the mRNA expression of immune response gene sets from high to low levels, promoting tumor immune escape and thus facilitating tumor progression (Fejzo et al., 2021). Here, our research findings reveal the significant overexpression of SOCS3 and FPR2 in the test set and validation set of patients with LUAD, as well as in mouse tumor cell lines, and these two genes are significantly enriched in exhausted T cells. To further elucidate the potential biological functions of KDM6B and KANSL1, we conducted a comprehensive Gene Set Enrichment Analysis (GSEA) at the transcriptome level. Surprisingly, the pathways in which these two genes are enriched exhibit a considerable overlap in patients with LUAD, such as Toll like receptor signaling pathway and JAK-STAT signalling pathway. Subsequently, *in vitro* experiments of CD8^+^T cells, it was found that, compared with the control group, these two genes significantly promoted the exhaustion of CD8^+^T cells. This suggests that these two genes may play an important role in the process of immune escape in LUAD. Although our study contributes to a deeper understanding of the roles and potential mechanisms of A-ERGs in LUAD, it also has some limitations. Firstly, our reliance on publicly available datasets may not fully capture the heterogeneity of different samples. Our findings are mainly based on bioinformatics analysis, which requires further experiments and validation with more clinical samples. Finally, while our study highlights the potential biological functions and related associations, the specific molecular mechanisms remain to be elucidated and verified.

## Conclusion

Our study, for the first time, adopted an analytical approach combining transcriptomics, single-cell transcriptomics, and cell experiments to explore the roles of A-ERGs in the occurrence and development of LUAD and their underlying molecular mechanisms. This research demonstrated that A-ERGs may regulate the occurrence of LUAD by mediating exhausted T cells, and analyzed two hub genes, namely KANSL1 and KDM6B, as diagnostic biomarkers for LUAD, thus providing new strategies and targets for potential therapies that could block the occurrence and metastasis of LUAD in the future.

## Data Availability

The datasets provided in this study can be found in an online repository. The repository names and accession numbers in the study are included in the article/supplementary materials.
