# Supplementary figures and images for: Revealing key regulatory factors in lung adenocarcinoma: the role of epigenetic regulation of autophagy-related genes from transcriptomics, scRNA-seq, and machine learning
Source: Front Pharmacol. 2025 Aug 4;16:1542338. doi: 10.3389/fphar.2025.1542338 (PMC12359839; doi:10.3389/fphar.2025.1542338)

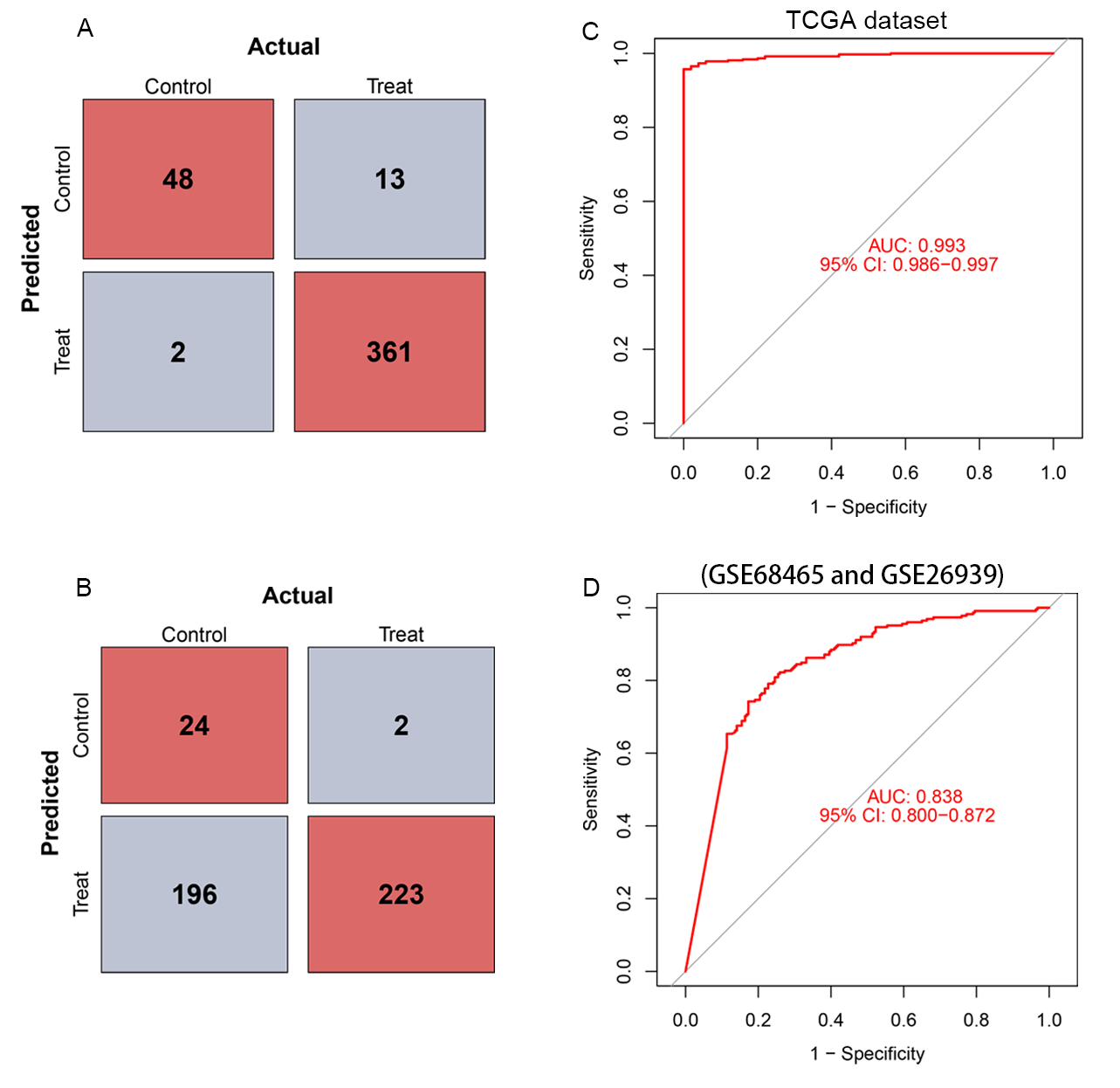

Supplement: Supplementary file 1 [file Image3.tif]

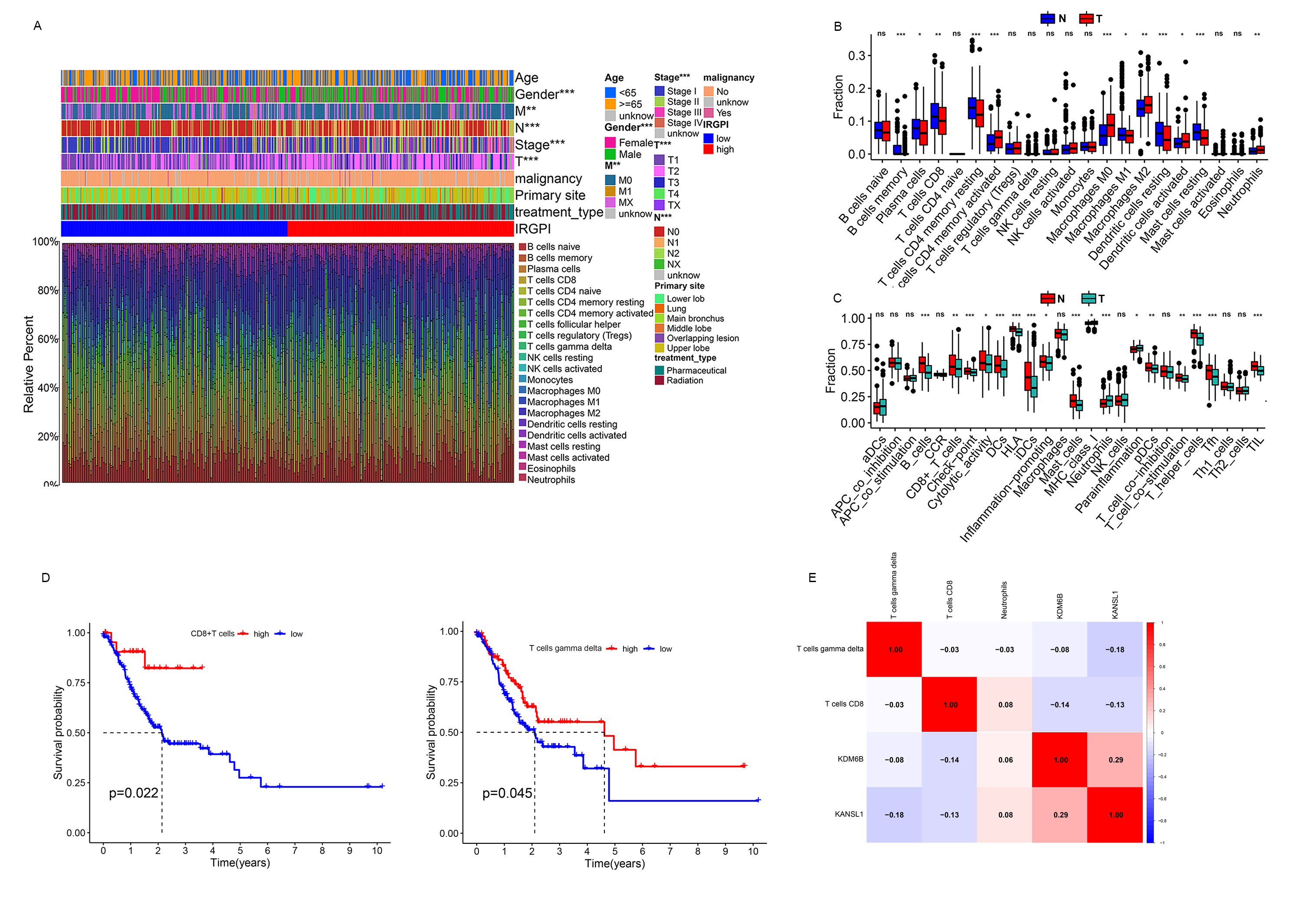

Supplement: Supplementary file 2 [file Image4.tif]

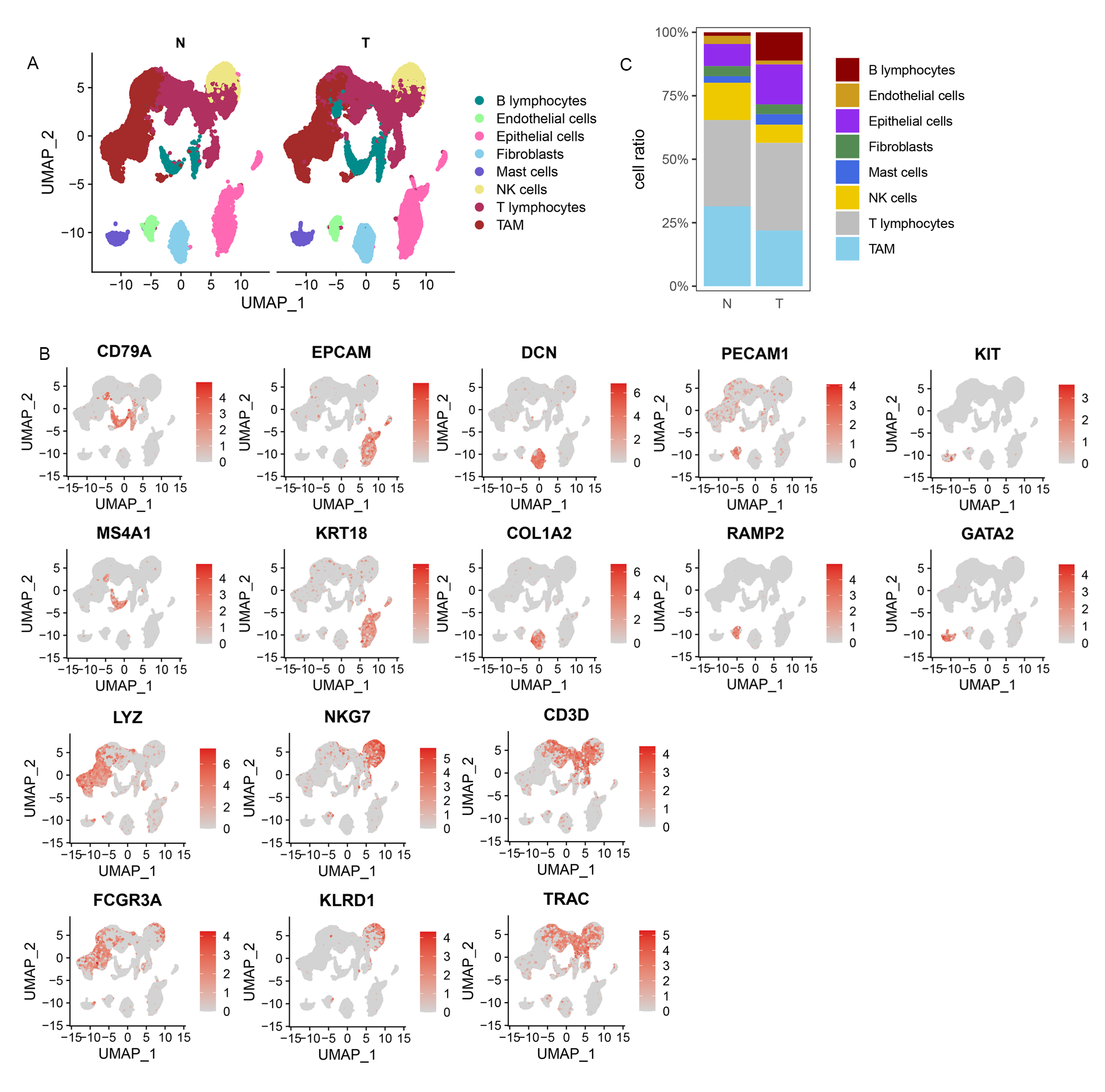

Supplement: Supplementary file 3 [file Image2.tif]

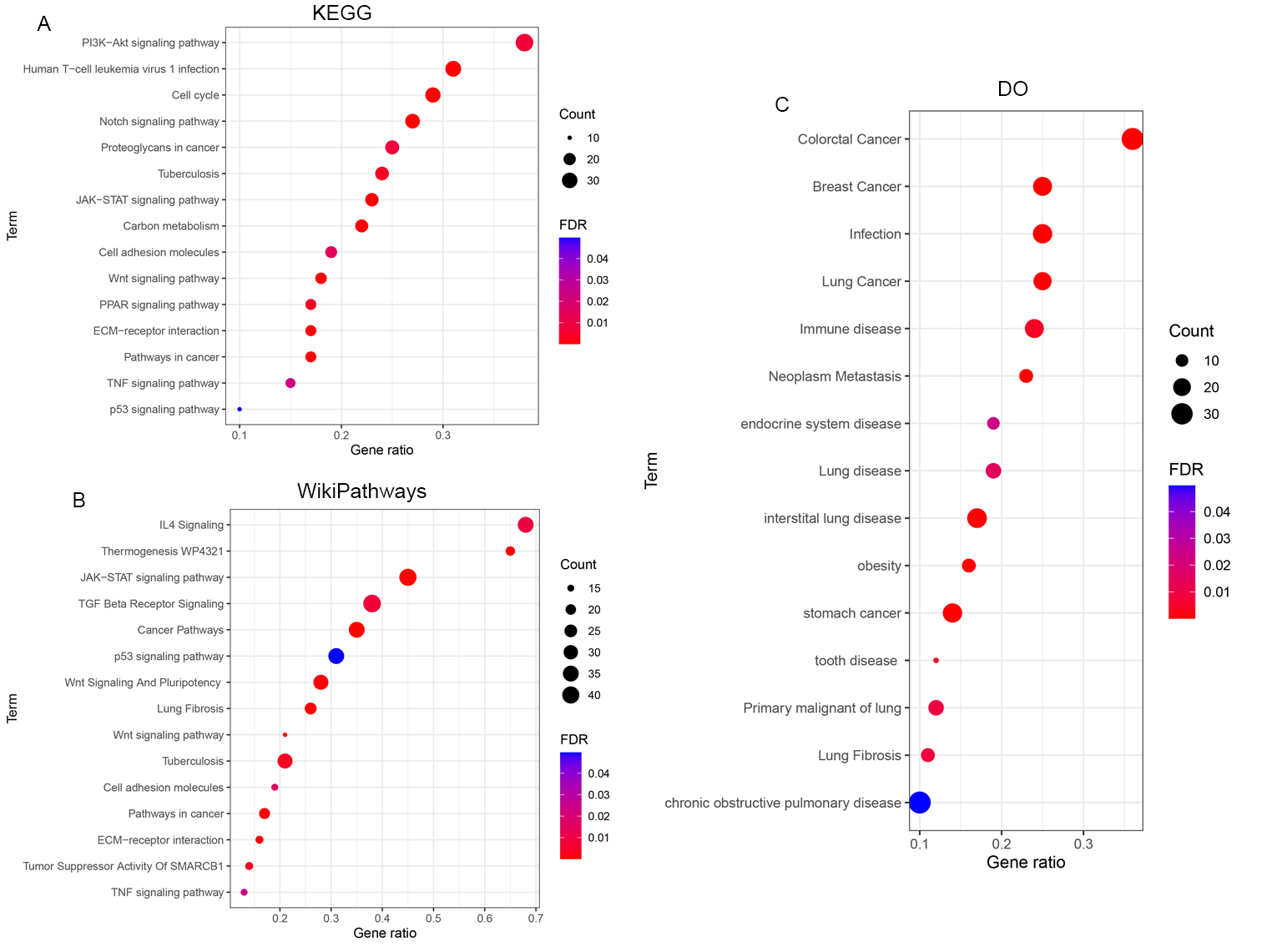

Supplement: Supplementary file 4 [file Image1.tif]

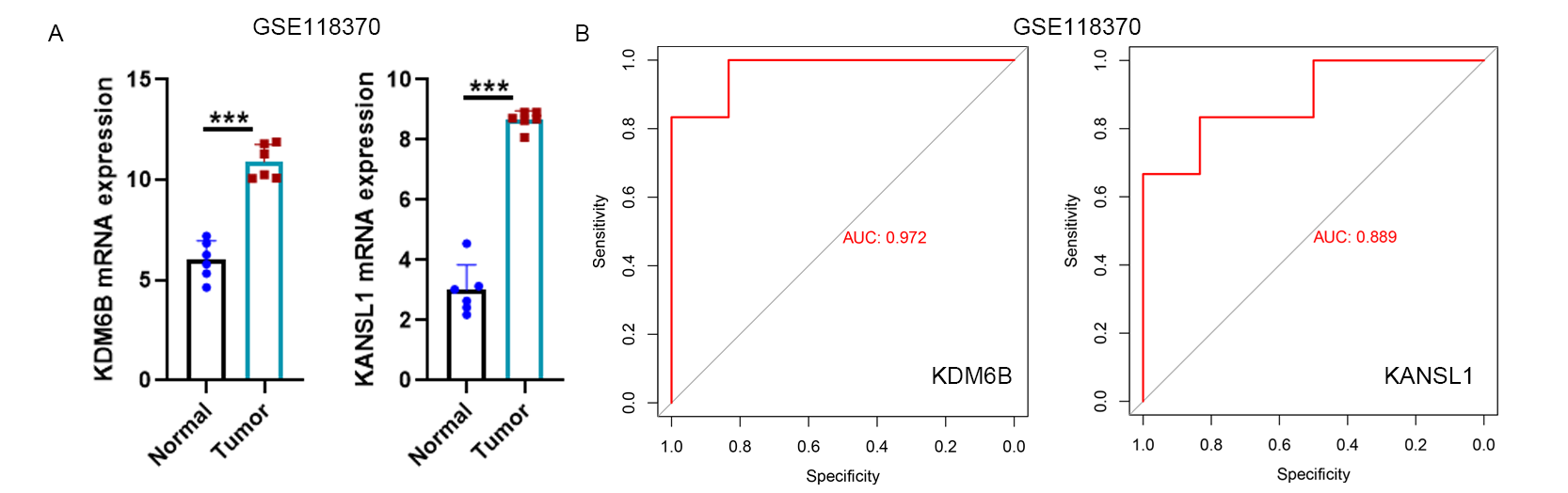

Supplement: Supplementary file 5 [file Image5.tif]
